# Program Spending to Increase Adherence: South African Cervical Cancer Screening

**DOI:** 10.1371/journal.pone.0005691

**Published:** 2009-05-28

**Authors:** Jeremy D. Goldhaber-Fiebert, Lynette A. Denny, Michelle De Souza, Louise Kuhn, Sue J. Goldie

**Affiliations:** 1 Program in Health Decision Science, Department of Health Policy and Management, Harvard School of Public Health, Boston, Massachusetts, United States of America; 2 Centers for Health Policy and Primary Care and Outcomes Research, Department of Medicine, Stanford University School of Medicine, Stanford, California, United States of America; 3 Gynaecology Oncology Unit, Institute of Infectious Disease and Molecular Medicine, University of Cape Town, Cape Town, South Africa; 4 Gertrude H. Sergievsky Center, College of Physicians and Surgeons, and Division of Epidemiology, Joseph L. Mailman School of Public Health, Columbia University, New York, United States of America; 5 Khayelitsha Cervical Cancer Screening Project (KCCSP), Cape Town, South Africa; University of Sydney, Australia

## Abstract

**Background:**

Adherence is crucial for public health program effectiveness, though the benefits of increasing adherence must ultimately be weighed against the associated costs. We sought to determine the relationship between investment in community health worker (CHW) home visits and increased attendance at cervical cancer screening appointments in Cape Town, South Africa.

**Methodology/Principal Findings:**

We conducted an observational study of 5,258 CHW home visits made in 2003–4 as part of a community-based screening program. We estimated the functional relationship between spending on these visits and increased appointment attendance (adherence). Increased adherence was noted after each subsequent CHW visit. The costs of making the CHW visits was based on resource use including both personnel time and vehicle-related expenses valued in 2004 Rand. The CHW program cost R194,018, with 1,576 additional appointments attended. Adherence increased from 74% to 90%; 55% to 87%; 48% to 77%; and 56% to 80% for 6-, 12-, 24-, and 36-month appointments. Average per-woman costs increased by R14–R47. The majority of this increase occurred with the first 2 CHW visits (90%, 83%, 74%, and 77%; additional cost: R12–R26).

**Conclusions/Significance:**

We found that study data can be used for program planning, identifying spending levels that achieve adherence targets given budgetary constraints. The results, derived from a single disease program, are retrospective, and should be prospectively replicated.

## Introduction

Health programs aimed at diseases such as HIV/AIDS, tuberculosis, type 2 diabetes, hypertension, and cancer, require individuals to return repeatedly to sites of clinical care in order to be effective [1]. As such programs generally have upfront costs and delayed benefits due to averted disease and death, interventions are undertaken to increase participation, return, and adherence to health recommendations [2]. Adherence interventions employ a broad range of methods (e.g., telephone reminders, home visits, and direct monetary incentives), rely on diverse theories, and achieve variable success [3], [4], [5], [6], [7], [8], [9].

Increasing adherence requires potentially substantial monetary expenditure beyond those needed to deliver the direct services of the health program. Understanding the relationship between this investment and the benefit of increased adherence is important when planning health care programs. In the planning phase, alternatives to population-based data must be sought to support decision making and budgeting prior to implementation.

In South Africa, cervical cancer is the leading cause of cancer and cancer mortality, a pattern still faced by developing countries worldwide – cervical cancer being one of the two leading causes of cancer death among women, with an estimated 270,000 deaths annually [10]. Patterns of cervical cancer differ between developed and developing countries, with the majority of cervical cancer burden occurring in developing countries, typically detected via symptoms at later invasive stages [11]. High-quality screening programs relying on repeated, periodic cervical Pap smears have led to population benefit in developed countries [12]. In most developing countries, the lack of organized cervical cancer screening means that reductions in cervical cancer mortality have yet to be achieved. While South Africa has instituted a screening policy in the public sector with the goal of providing screening at age 30, 40, and 50 to 70% of women, current coverage and follow-up do not meet these goals [13], [14]. In addition to Pap smears, the effectiveness of a variety of other screening technologies, with fewer clinic visits between screening and treatment, has been studied [15], [16] as have two human papillomavirus (HPV) vaccines that prevent infection with viral subtypes responsible for the majority of cervical cancers [17], [18]. Because both vaccines are prophylactic, they are ideally used prior to sexual debut. As such, even with a choice to vaccinate teenage girls now which would require overcoming barriers like affordability, delivery mechanisms for adolescents, and uncertainty about the long-term benefits, policymakers must still make important decisions regarding screening to provide direct benefits to women currently in their 30s and 40s.

To support decision making relating to cervical cancer screening in South Africa, this analysis focuses on the relationship of monetary expenditure and a key element of prevention effectiveness – adherence – in the context of a large community-based screening program in resource poor, peri-urban areas near Cape Town. Our analysis extends previously published work [19] that quantified the resources used to conduct unlimited community health worker (CHW) home visits and associated levels of screening appointment attendance. For our present analysis, we increased both the quantity and range of the data previously examined, widening the window of appointments covered from 1 to 2 years and extending longitudinal follow-up from 2 to 3 years. Motivated to generate useful information for program planning and cost-effectiveness analyses, we illustrate a method for estimating how choosing intermediate levels of CHW program intensity, expressed in monetary terms, translates into expected changes in appointment attendance.

## Methods

### Study setting and population

The screening program and community health worker (CHW) home visit program we analyze here have been described previously [19], [20]. Briefly, in the context of the program, a study was conducted to evaluate cervical cancer screening alternatives at multiple clinic sites in a largely poor, black peri-urban area in which 60% of households are part of informal settlements without full electric, sewage, and road services. Women age 35–65 years were recruited from the community via posters placed in community health centers and a variety of community-based outreach programs that included presentations at churches, community meetings, and radio programs. Cervical cancer screening was performed at 3 sites on the grounds of existing health facilities. The screening study enrolled 7,000 eligible women, approximately 10–15% of the 50,000–70,000 women age 35–65 living in Khayelitsha [21], [22].

The screening study was approved by the institutional review boards of Columbia University (New York, NY) and the University of Cape Town (Cape Town, South Africa). All participants provided written informed consent. The study was conducted according to the principles expressed in the Declaration of Helsinki.

Women in the screening study have a series of scheduled follow-up appointments (1-month, 6-month, 12-month, 24-month, and 36-month) in which they receive cervical cancer screening tests. Women testing positive receive diagnostic colposcopy and biopsy as well as treatment and referral based on the results of these diagnostic tests. For women who do not return spontaneously for their scheduled appointments, groups of CHWs drive through the community making home visits to reschedule appointments and encourage attendance, with CHW visits repeated until return is achieved or further participation is declined. Women participating in the screening study with 6-, 12-, 24-, or 36-month appointments scheduled in 2003–4 were considered eligible for inclusion in this analysis.

### Cost data

We estimated the cost of the entire CHW home visit program which made visits both to women enrolled in the study and to other women receiving services from the larger screening program. We adopted an ingredients-based approach to costing. We identified the types of resources used: 1) CHW time spent driving and making home visits, 2) fuel used during CHW visits, 3) maintenance and depreciation of the vehicles. For each resource type, we then assigned a monetary value for a unit quantity. Finally, the number of units used and unit values were multiplied and then summed to produce a total cost estimate. All costs are presented in 2004 Rand (R) with prices from other years adjusted to 2004 levels based on the South African Gross Domestic Product deflator [23].

In the program, CHW home visits were grouped into trips, with a trip being defined as leaving the clinic, driving a circuit through the community making multiple home visits, and returning to the clinic. The total amount of CHW time per home visit equaled the number of CHWs on the trip multiplied by the duration of the trip divided by the number of home visits. CHW time was valued at R2,500 per month for a 1,840 hour work year (R16.3 per hour). Fuel and vehicle maintenance costs were derived directly from the project's cost accounting system. Vehicle depreciation was based on purchase price, excluding taxes and duties, and used a five-year straight-line formula assuming a 3% discount rate and no resale value [19]. As program vehicles are used for other purposes, we estimated that 50% of usage was attributable to CHW home visits. All fuel, maintenance and vehicle depreciation costs were, therefore, multiplied by 50%. The total CHW home visit cost was divided by the number of CHW home visits conducted to produce a cost per home visit which could then be applied to those CHW visits associated with women enrolled in the screening study in subsequent analyses.

### Appointment data

Attendance for scheduled appointments was classified into three categories: 1) spontaneous, on-time return; 2) late return with CHW visit(s); 3) no return despite CHW visits. Two sources of data were used to classify appointment attendance. First, the study appointments database indicated the date when individuals attended appointments compared to their scheduled dates. Second, weekly CHW summary logbooks were transcribed and verified against individual CHW visit reports to create an electronic database of CHW home visits. By linking the databases, we identified on-time versus late attendance and how many CHW visits preceded appointment attendance.

### CHW spending limits and attendance

In the study, CHW home visits continued until a woman returned or declined further participation. In addition to analyzing the cost of CHW visits relative to return rates as observed, we considered hypothetical alternative policies for CHW visits. Specifically, we asked how much would be spent and what level of appointment attendance achieved if each woman could receive no more than a fixed number of CHW visits for a given appointment type. For this analysis, we assumed that CHW visits caused additional appointment attendance and that those women who did not return after receiving the maximum number of CHW visits allowed under a given policy would not return spontaneously afterwards.

Under these assumptions, we constructed response functions relating spending (the independent variable) to appointment attendance (the dependent variable) via CHW visits. For each appointment type, we sequentially imposed hypothetical policies where the maximum number of CHW visits was set at higher and higher levels. For each policy, we used the observed data to estimate the additional percentage of all women eligible for that appointment type who returned with no more than the maximum number of CHW visits allowed by the policy. Based on the number of additional CHW visits performed under the policy, we also calculated the additional cost. Taken together, these quantities formed the response functions for each appointment type. Each function's input was spending per woman on CHW visits with the output being the percentage of women who returned for their appointments.

### Statistical analyses

Intercooled Stata 8.2 (Stata Corporation, College Station, Texas, USA) was used for all statistical analyses. Linking and classification of study databases and CHW logbook databases were performed using Excel 2003's Visual Basic for Applications (Microsoft Corporation, Redmond, Washington, USA).

## Results

In 2003–4, 981 CHW trips were conducted. The mean number of women visited per trip was 5.4 (standard deviation: 2.3, range: 1–18), and the mean number of CHWs who went on each visit was 2.9 (standard deviation: 0.7; range: 2–5). The mean time spent visiting each woman during a trip was 21.9 minutes (standard deviation 13.6; range: 3–150). This implies that the 5,258 CHW visits conducted required 5,566 hours of CHW time driving throughout the community and visiting women in their homes.

Over the 2 year period, the value of CHW time spent making home visits was R91,399, while fuel, vehicle maintenance, and depreciation costs attributable to CHW visits were R37,704, R28,130, and R36,785, respectively. In total, the CHW home visits cost R194,018 with a cost per CHW home visit of R36.6.

Of the 5,258 CHW visits conducted, 4,617 visits were made for women enrolled in the study, identified via the study database linkage, with the remainder targeting women receiving screening services through the program but not enrolled in this study. Subsequent results are reported solely for enrolled women.

Table 1 shows that 74%, 55%, 48%, and 56% of women attended their scheduled 6, 12, 24, and 36 month appointments spontaneously and without CHW visits.

**Table 1:** Relationship of attendance and CHW visits* ^,^ **.

	6 month (n = 1,003)		12 month (n = 1,992)		24 month (n = 1,928)		36 month (n = 647)	
Visit Policy***	# Attending	CHW Visits	# Attending	CHW Visits	# Attending	CHW Visits	# Attending	CHW Visits
0	740	0	1,098	0	944	0	453	0
1	892	201	1,559	533	1,322	532	575	182
2	906	305	1,650	817	1,448	1,044	624	356
3	907	326	1,702	1,153	1,490	1,440	640	515
4	907	338	1,720	1,333	1,508	1,684	645	595
5	–	–	1,729	1,523	1,513	1,809	646	635
6	–	–	1,735	1,613	1,517	1,899	646	647
7	–	–	1,739	1,655	1,517	1,920	646	647
8	–	–	1,739	1,671	1,518	1,928	647	671
9	–	–	1,739	1,680	–	–	–	–

* CHW – community health worker.

** The counts of the number of women attending appointments and the number of CHW visits made are both cumulative.

*** Visit policy defined as no more than ‘x’ CHW visits per woman per appointment type.

**Figure 1:**
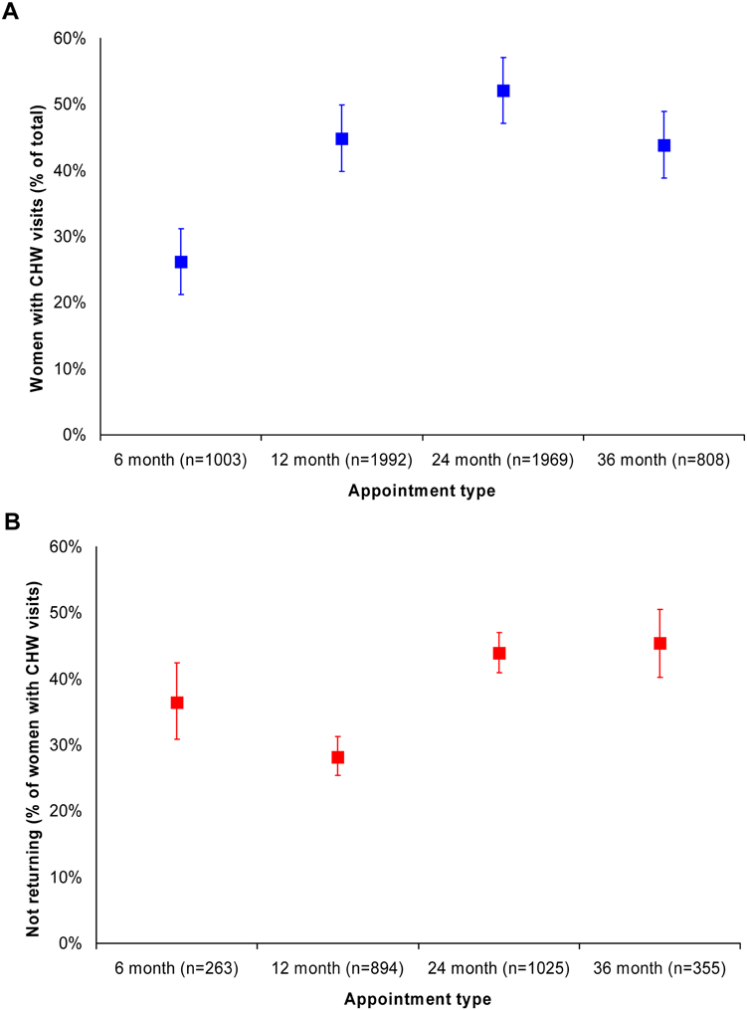
CHW visits and patient failure to return. Shown in the graph are the percentage and 95% confidence intervals of women who received 1 or more CHW visits by appointment type during 2003–4 (Panel A, blue squares); and the percentage and 95% confidence intervals of women receiving CHW visits who did not attend their scheduled appointments despite those visits (Panel B, red squares).

Figure 1, Panel A shows the percentages of women who required 1 or more CHW visits for each appointment type. In general, the percentage of women who did not return for their scheduled appointments spontaneously increased for subsequent appointment types. While the 36 month appointment had a somewhat lower percentage of women requiring CHW visits than for the 12 and 24 month appointments, the 12, 24, and 36 month appointment types all had significantly higher percentage of woman requiring CHW visits than did the 6 month appointment. Figure 1, Panel B shows the percentage of women requiring 1 or more CHW visits who never returned despite those CHW visits. Failure to return despite CHW visits is greater in the 24 and 36 month appointments than in the 12 and 6 month points.

The number of CHW visits required for each appointment type was: 338, 1,680, 1,928, and 671 for the 6, 12, 24, and 36 month appointments, respectively (Table 1). The total additional cost per woman ultimately attending these appointment types increased for subsequent appointments and was: R13.6, R35.4, R46.6, and R38.0, respectively.

**Figure 2:**
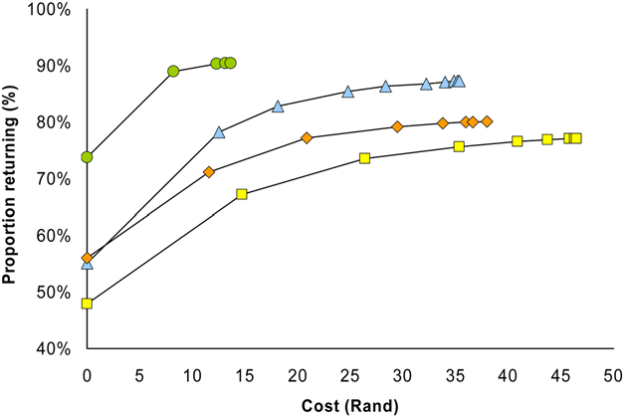
Relationship between increased attendance and additional cost. Shown in the graph are the response functions relating the additional cost of policies allowing greater number of CHW home visits per woman to increases in appointment attendance. Separate functions are shown for 6-month appointments (green circles), 12-month appointments (blue triangles), 24-month appointments (yellow squares), and 36-month appointments (orange diamonds).

Figure 2 and Table 1 show the relationship between increasing the number of women attending appointments after CHW home visit(s) and the increased cost associated with those home visits by appointment type. Attendance without CHW visits is nearly 75% for the 6-month appointment type but is between 48% and 56% for the 12-, 24-, and 36-month appointments. After 2 CHW visits, 6-month attendance increased to 90% of appointments at a cost of R12.3. After 2 CHW visits, 12-, 24-, and 36-month attendance increased to 83%, 74%, and 77% at costs of R18.1, R26.4, and R21.0 respectively. For these latter appointment types, using a policy of no more than 2 CHW home visits per woman per appointment type resulted in at least 95% of women who ever returned attending their appointments at less than 60% of the cost of making CHW visits until a woman returned or declined further participation. For the 6-month appointment, similar results were achieved if the policy was having no more than 1 CHW home visit per woman per appointment.

## Discussion

In the context of this South African cervical cancer screening program, high levels of repeated attendance (>75%) were achieved at scheduled screening appointments using community health worker (CHW) home visits. For follow-up appointments scheduled 1 to 3 years after initial screening, CHW home visits represented an appreciable additional cost (above R35 per woman screened). As cervical cancer prevention programs that achieve high adherence can be both effective and cost-effective [24], [25] and thereby justify public health spending, we examined potential efficiencies within CHW efforts to maintain adherence. By evaluating the patterns of CHW visits and women's subsequent attendance at their screening appointments, we found the vast majority of additional attendance (>95%) occurred with no more than 2 CHW visits per woman per appointment type, requiring approximately 60% or less of the cost of the unlimited CHW visit program.

An important limitation is the generalizability from this study to current South African cervical cancer screening policy. While our study evaluates participant adherence over a 3 year follow-up period, current South African recommendations involve screening at 10 year intervals (at age 30, 40, and 50 years). In our study, adherence without intervention declined as the interval between screening appointments lengthened, and it is quite possible that the level of adherence after 10 years would be very low. Such low adherence would certainly require some intervention. However, it could be that the effectiveness of a CHW-based adherence program after 10 years would be low or simply be more costly given the large portion of screened individuals that might require CHW visits. Interestingly, Goldie et al. find that using screening intervals of 5 years (age 35, 40, and 45 years) appears more effective and cost-effective than screening every 10 years (age 30, 40, and 50 years) in South Africa [26]. Combining this finding with the possibility of lower adherence over longer screening intervals, 5 year screening intervals may be even more attractive.

While the costs and adherence observed in the context of the study accurately reflect the experience in one peri-urban center in South Africa, other locations and interventions may result in different costs and impact of CHW visits on adherence. For example, in a pre-post community-based study, the use of intensive pre-counseling by CHWs was associated with an increase of more than 50% in the proportion of women choosing voluntary counseling and testing for HIV, compared to shorter, standard nurse-provided counseling. Similarly, it was associated with increases in self-reported consistent condom use over 1 and 6 month periods (increased 10% and 5% respectively) (Lynette Denny, University of Cape Town, unpublished data, 2009). In another local program providing CHW home visits and basic nursing care for people living with HIV/AIDS, orphans, and the elderly [27], the use of bicycles by 175 CHWs was expected to increase their average number of home visits conducted from 6–8 to 12–16 per day with the bicycles costing approximately $300/bicycle (2008 USD) and projected to have useful lives of 5–10 years (Amanda Zar, MaAfrika Tikkun, personal communication, 2009). In such programs, the costs and effectiveness associated with the choice to implement a CHW program and the intensity with which to equip and utilize the program could be evaluated using the method described in this study. As the use of CHWs has expanded at a national level in South Africa [28], such future studies could be used to assess the generalizability of our findings.

We note that alternatives to CHW home visits such as cell phone-based interventions were not tested in the study, though, in theory, they could be more effective and/or less costly than CHW visits. While phone ownership was quite low in our sample of 35–65 year old women, cell phone subscriptions have nearly tripled in South Africa since 2003, making the use of cell phones an important feature for a variety of services offered to relatively resource poor individuals in South Africa and elsewhere [23], [29]. In some contexts, the ease with which cell phone plans are switched and phone numbers changed as well as the frequency of cell phone thefts leave open the question of the most effective means of maintaining long-term contact to deliver healthcare services.

It is important to emphasize that the response functions relating costs due to CHW visits and appointment attendance are based on hypothetical policies of making no more than a given number of CHW visits for a given woman and appointment type. Such policies were not prospectively tested in the screening population to produce observed counterfactuals. However, the patterns of return for appointments across appointment types are plausible – earlier appointments with shorter time periods between clinical contacts (6-month and 12-month appointments) have greater spontaneous attendance and/or greater responsiveness to the first CHW home visit compared to 24- and 36-month appointments (Figure 2). Additionally, appointment attendance levels after CHW home visits are also broadly similar for the 24- and 36-month appointment types, suggesting the emergence of more stable longer term usage patterns.

Adherence response functions can be used in a variety of ways. First policymakers can calculate how, under a fixed budget, different intervals between clinical contacts may lead to different rates of appointment attendance. Alternatively, for a target level of appointment attendance, the associated cost can be computed. For model-based cost-effectiveness analyses, levels of adherence are entered as a parameter based on initial assumptions. The impact of the adherence parameter on model-simulated outcomes is typically explored via sensitivity analyses in which the level of adherence is varied systematically. However, as we demonstrate, different levels of adherence can be achieved through different, non-linear levels of program expenditure, and thus sensitivity analyses of adherence could be improved by simultaneously varying the level of adherence and its associated costs based on response functions like those we have estimated here.

Substantial improvements in appointment attendance can be achieved in a South African cervical cancer screening context using community health worker home visits. As home visits require money and resources that could be used to provide other healthcare services, seeking to maximize attendance while controlling expenditure is both important and prudent. In this context, we illustrate a method for estimating the relationship between monetary investment in subprograms focusing on adherence and observed increases in adherence, using data from a longitudinal, community-based demonstration project. Such an approach may be useful for public health program planning and budgeting across a broad range of diseases and country settings.

## References

[1] Beaglehole R, Epping-Jordan J, Patel V, Chopra M, Ebrahim S (2008). Improving the prevention and management of chronic disease in low-income and middle-income countries: a priority for primary health care.. Lancet.

[2] Cleemput I, Kesteloot K (2002). Economic implications of non-compliance in health care.. Lancet.

[3] Hirsch-Moverman Y, Daftary A, Franks J, Colson PW (2008). Adherence to treatment for latent tuberculosis infection: systematic review of studies in the US and Canada.. Int J Tuberc Lung Dis.

[4] Liu Q, Abba K, Alejandria MM, Balanag VM, Berba RP (2008). Reminder systems and late patient tracers in the diagnosis and management of tuberculosis.. Cochrane Database Syst Rev.

[5] Roter DL, Hall JA, Merisca R, Nordstrom B, Cretin D (1998). Effectiveness of interventions to improve patient compliance: a meta-analysis.. Med Care.

[6] Stirratt MJ, Gordon CM (2008). Adherence to biomedical HIV prevention methods: considerations drawn from HIV treatment adherence research.. Curr HIV/AIDS Rep.

[7] van Dulmen S, Sluijs E, van Dijk L, de Ridder D, Heerdink R (2007). Patient adherence to medical treatment: a review of reviews.. BMC Health Serv Res.

[8] Volmink J, Garner P (2000). Interventions for promoting adherence to tuberculosis management.. Cochrane Database Syst Rev.

[9] Yabroff KR, Kerner JF, Mandelblatt JS (2000). Effectiveness of interventions to improve follow-up after abnormal cervical cancer screening.. Prev Med.

[10] Parkin DM, Bray F, Ferlay J, Pisani P (2005). Global cancer statistics, 2002.. CA Cancer J Clin.

[11] Parkin DM, Bray F (2006). Chapter 2: The burden of HPV-related cancers.. Vaccine.

[12] Kitchener HC, Castle PE, Cox JT (2006). Chapter 7: Achievements and limitations of cervical cytology screening.. Vaccine.

[13] National Guidelines on Cervical Cancer: Screening Programme.

[14] Denny L (2006). Cervical cancer: the South African perspective. FIGO 6th Annual Report on the Results of Treatment in Gynecological Cancer.. Int J Gynaecol Obstet.

[15] Arbyn M, Sasieni P, Meijer CJ, Clavel C, Koliopoulos G (2006). Chapter 9: Clinical applications of HPV testing: a summary of meta-analyses.. Vaccine.

[16] Denny L, Quinn M, Sankaranarayanan R (2006). Chapter 8: Screening for cervical cancer in developing countries.. Vaccine.

[17] Koutsky LA, Harper DM (2006). Chapter 13: Current findings from prophylactic HPV vaccine trials.. Vaccine.

[18] Schiller JT, Castellsague X, Villa LL, Hildesheim A (2008). An update of prophylactic human papillomavirus L1 virus-like particle vaccine clinical trial results.. Vaccine.

[19] Goldhaber-Fiebert JD, Denny LE, De Souza M, Wright TC, Kuhn L (2005). The costs of reducing loss to follow-up in South African cervical cancer screening.. Cost Eff Resour Alloc.

[20] Denny L, Kuhn L, De Souza M, Pollack AE, Dupree W (2005). Screen-and-treat approaches for cervical cancer prevention in low-resource settings: a randomized controlled trial.. JAMA.

[21] (2006). http://www.capegateway.gov.za/other/2007/10/kprufinal_2005_october_2007_publish_date.pdf.

[22] (2001). http://www.statssa.gov.za/census01/HTML/default.asp.

[23] (2005). World Development Indicators.

[24] Goldie SJ, Kuhn L, Denny L, Pollack A, Wright TC (2001). Policy analysis of cervical cancer screening strategies in low-resource settings: clinical benefits and cost-effectiveness.. JAMA.

[25] Goldie SJ, Gaffikin L, Goldhaber-Fiebert JD, Gordillo-Tobar A, Levin C (2005). Cost-effectiveness of cervical-cancer screening in five developing countries.. N Engl J Med.

[26] Goldie SJ, Gaffikin L, Goldhaber-Fiebert JD, Gordillo-Tobar A, Levin C (2005). Supplement to: Cost-effectiveness of cervical-cancer screening in five developing countries.. N Engl J Med.

[27] (2008). Bicycling Bike Town assists MaAfrika Tikkun.

[28] Schneider H, Hlophe H, van Rensburg D (2008). Community health workers and the response to HIV/AIDS in South Africa: tensions and prospects.. Health Policy Plan.

[29] Shackleton S-J (2007). Rapid Assessment of Cell Phones for Development.

